# Development and validation of a lipogenic genes panel for diagnosis and recurrence of colorectal cancer

**DOI:** 10.1371/journal.pone.0229864

**Published:** 2020-03-10

**Authors:** Ehsan Gharib, Parinaz Nasrinasrabadi, Mohammad Reza Zali

**Affiliations:** 1 Basic and Molecular Epidemiology of Gastrointestinal Disorders Research Center, Research Institute for Gastroenterology and Liver Diseases, Shahid Beheshti University of Medical Sciences, Tehran, Iran; 2 Department of Molecular Medicine, Institute of Medical Biotechnology, National Institute of Genetic Engineering and Biotechnology, Tehran, Iran; 3 Gastroenterology and Liver Diseases Research Center, Research Institute for Gastroenterology and Liver Diseases, Shahid Beheshti University of Medical Sciences, Tehran, Iran; Osaka Medical Center for Cancer and Cardiovascular Diseases, JAPAN

## Abstract

**Background & aim:**

Accumulated evidence indicates that the elevation of lipid metabolism is an essential step in colorectal cancer (CRC) development, and analysis of the key lipogenic mediators may lead to identifying the new clinically useful prognostic gene signatures.

**Methods:**

The expression pattern of 61 lipogenic genes was assessed between CRC tumors and matched adjacent normal tissues in a training set (n = 257) with the Mann-Whitney U test. Cox's proportional hazards model and the Kaplan–Meier method were used to identifying a lipogenic-biomarkers signature associated with the prognosis of CRC. The biomarkers signature was then confirmed in two independent validation groups, including a set of 223 CRC samples and an additional set of 203 COAD profiles retrieving from the Cancer Genome Atlas (TCGA).

**Results:**

Five genes, including ACOT8, ACSL5, FASN, HMGCS2, and SCD1, were significantly enhanced in CRC tumors. Using the cutoff value 0.493, the samples were classified into high risk and low risk. The AUC of panel for discriminating of all, early (I-II stages), and advanced CRC (III-IV stages) were 0.8922, 0.8446, and 0.9162 (Training set), along with 0.8800, 0.8205, and 0.7351 (validation set I), and 0.9071, 0.8946, and 0.9107 (Validation set II), respectively. There was a reverse correlation between the high predicted point of panel and worse OS of CRC patients in training set (HR (95% CI): 0.1096 (0.07089–0.1694), *P* < 0.001), validation set I (HR (95% CI): 0.3350 (0.2116–0.5304), *P* < 0.001), and validation set II (HR (95% CI): 0.1568 (0.1090–0.2257), *P* < 0.001).

**Conclusion:**

Our study showed that the panel of ACOT8/ACSL5/FASN/HMGBCS2/SCD1 genes had a better prognostic performance than validated clinical risk scales and is applicable for early detection of CRC and tumor recurrence.

## Introduction

According to the global statics, Colorectal cancer (CRC) is currently ranked as the second and third most current cancers in women and men, respectively [1]. CRC population is growing about a million new cases annually, and nearly half of this number will die during the next five years. The highest rate of CRC incidence has been reported in developed countries, including Australia, the United States of America, Canada, etc., [2]. Although CRC prevalence in Iran is not high and mostly reported in middle-aged people, the later investigations indicate a growing trend of CRC in the younger population [3–5].

CRC mortality could be avoided if cancer is being diagnosed at the early stages. Therefore, the staging of tumors is an essential step in CRC progression. Treating of the advanced CRC cases with high dysplasia and the invasive lesion is mostly accompanied by failure [6, 7]. On the other hand, about one-third of stage II CRC patients are accounted for relapse within five years after tumor resection and died because of metastasis [8]. Consequently, several investigations have been carried out to identify novel biomarkers for improving CRC progression [5, 8].

Accumulated evidence indicates that the enhanced level of lipid metabolism has a crucial role in cancer development. Due to high proliferation activity, cancerous cells tend to supply their needed lipids *de novo*, which requires an abnormal level of lipogenic enzymes and signaling factors [9, 10]. So, analyzing the differences between the expression pattern of lipid-metabolic mediators in healthy and tumoral cells could be considered as a critical hallmark [11]. Thus, the current investigation was designed to develop a diagnostic panel based on the dysregulation of the lipogenic genes for early detection of CRC and tumor recurrence.

## Material and methods

### Ethical approval

This study was conducted in accordance with the ethical principles of the World Medical Association’s Declaration of Helsinki and approved by the Medical Ethical Committee of RIGLD, Tehran, Iran (Ethical code number: (IR.RIGLD.1397-947). Written informed consents were obtained from all study subjects or their parent/legal guardian in case of under 18 years old. All methods were carried out in accordance with relevant guidelines.

### Identification of lipid-metabolic related genes

The core list of lipid-metabolic related genes was retrieved using the Cytoscape plugin DisGenNet, a bioinformatics platform that integrates the genes data of various human disorders [12]. Following query terms were chosen as data sources: 1- Online Mendelian Inheritance in Man (OMIM) (Mendelian Inheritance in Man and its online version, OMIM), 2- Genetic Association Database (GAD) [13], 3- Mouse Genome Database (MGD) [14], 4- Comparative Toxicogenomics Database (CTD) [15], 5- PubMed, and 6- Uniprot. Genes were ranked according to the number of sources, organism type, and the number of supported publications.

### Clinical specimens

This investigation was conducted according to the recommendations of the ethics committee of the Research Institute for Gastroenterology and Liver Disease (RIGLD). The study population consisted of 300 formalin-fixed and paraffin-embedded (FFPE) tumor specimens and 300 matched adjacent normal tissues from CRC patients who underwent tumor resection at Shohadaye Tajrish Hospital, Tehran—Iran, during 2009–2015, and 180 CRC tumors and 180 matched adjacent normal tissues from the Taleghani Hospital, Tehran—Iran, during 2011–2017. Subjects median age was 52 years (range: 37–79 years). Clinical records and written informed consents were retrieved from the archive departments. Tumors assessment was done based on the American Joint Committee on Cancer (AJCC) guidelines [16]. Following ups were updated until March 2019 (average 1,286 ± 14.9 days, range 128–2411 days). Those patients who did not undergo preoperative chemotherapy or radiotherapy were chosen for this study.

### RNA extraction and cDNA synthesis

RNA extraction from the FFPE samples was carried out using the RNeasy FFPE Kit (QIAGEN, USA), based on the company’s guidelines. Genomic DNA contamination was avoided after one-hour treatment with the DNase I enzyme. The quality of RNA samples was assayed at the A260/A280 nm ratio using a spectrophotometer (Nanodrop Technologies, USA). The first strand cDNAs were synthesized with the QuantiTect Rev transcription kit (QIAGEN).

### Realtime-PCR

The amplification of target genes was carried out using the QuantiTect SYBR® Green PCR Kit (Qiagen, USA). The Realtime-PCR was performed using a LightCycler rapid thermal cycler (Roche, USA). The thermocycler was programmed as a 95°C for 15 min denaturation step, and 35 cycles consisted of 94°C for 15 s, 55°C for 30 s, and 72°C for 30 s. The expression level of target genes was normalized to snoRNU6 as an endogenous control gene, using the 2^-ΔΔCT^ method and analyzed with the relative expression software tool (REST, Qiagen, USA).

### TCGA mining and data processing

Illumina Hiseq 2000 RNA-seq colon cancer adenocarcinoma (COAD) dataset (level 3 per-gene RNA-seq v2 expression data) were downloaded from The Cancer Genome Atlas (TCGA, https://cancergenome.nih.gov). The read counts were estimated by RSEM package (RNA-seq by expectation maximization). The Coding‐Noncoding‐Index version 2 algorithms [17], as well as coding potential calculator‐2 web server [18], Pfam‐scan version 1.3 [19], and the phylogenetic codon substitution frequency [20], were used for the identification of protein-coding genes from the reads longer than 100 nucleotides. Detection of human transcription factors and kinases was done using the Ensembl BioMart web process version 79 (https://www.ensembl.org/Biomart) according to the human genome assembly GRCh38.p12. Genes quantitation was performed with the Cufflink software version 1.3.0, and Cuffdiff package version 2.2.1 was used to calculate the expression score of transcripts with the following formula: RPKM = total exon reads/mapped reads in millions × exon length in kb (RPKM = reads per kilobase of transcript per million mapped reads). The false discovery rate (FDR) was set as %5, and the q‐value (p‐adjusted) was given as <0.05.

### Statistical analysis

Statistical analysis was carried out with the IBM SPSS Statistics software version 22 (IBM, USA). The Chi-square test and unpaired unequal variance t-test were performed to compare the variables between the target groups. Mann-Whitney U test was used for the analysis of the level of the genes. Spearman test was employed to analyze the relationship between the differential expression of the target RNAs and clinicopathologic characteristics. The receiver operating characteristic (ROC) curve was drawn to evaluate the value of the selected genes in the diagnosis of CRC. Cox’s proportional hazards model was used for univariate and multivariate logistic regression. The goodness of fit of the multivariate models was calculated with the Hosmer–Lemeshow test. Kaplan–Meier survival was applied to estimate patients 5-year overall survival (OS). All data are represented as the mean ± S.D. (Standard deviation) and taken as significance if *P* < 0.05 (*).

## Results

### Patients descriptive

The study group consisted of 480 CRC tumor specimens along with their matched adjacent normal tissues, including 272 men and 208 women. Among them, 237 patients (49.37%) were detected with early CRC (I-II TNM stage), and 243 patients (50.63%) were grouped in advanced CRC (III-IV TNM stage). Patients were subsequently divided into a training set (257 CRC tumors and matched normal samples), and validation set I (223 CRC tumors and matched normal samples). Additionally, an independent validation set II consisted of 253 TCGA-COAD profiles, including 203 patients (113 early and 90 advanced CRC) along with 50 healthy individuals was also considered for panel analysis. These target sets were statistically different based on the clinical variables. Additional details are demonstrated in Table 1.

**Table 1:** Clinical features of the studied population.

Variable	Training set (%)	Validation set I (%)	Validation set II (%)	*p* value
**Healthy count (%)**				
** Sex**				0.463
** Male**	136 (52.9)	136 (60.99)	34 (52.9)	
** Female**	121 (47.1)	87 (39.01)	16 (47.1)	
** Age (year)**				0.877
** Mean + SD**	53 ± 3	51 ± 2	51 ± 4	
**Colorectal cancer count (%)**				
** Sex**				0.192
** Male**	136 (52.9)	136 (60.99)	119 (58.62)	
** Female**	121 (47.1)	87 (39.01)	84 (41.38)	
** Age (year)**				0.801
**Mean + SD**	65 ± 3	65 ± 2	65 ± 2	
**Location of tumor**				0.122
** Left Colon**	93 (36.19)	79 (35.43)	77 (37.94)	
** Right Colon**	86 (33.46)	102 (45.74)	82 (40.39)	
** Rectosigmoeid**	78 (30.35)	42 (18.83)	44 (21.67)	
**Differentiation**				0.073
** Well**	59 (22.96)	51 (22.87)	62 (30.54)	
** Moderately**	97 (37.74)	88 (39.46)	77 (37.93)	
** Poorly**	101 (39.30)	84 (37.67)	64 (31.53)	
**Lymph node Metastasis**				0.209
** Yes**	81 (31.52)	73 (32.74)	123 (60.60)	
** No**	176 (68.48)	150 (67.26)	80 (39.40)	
**TNM stage**				0.103
** I**	60 (23.35)	46 (20.63)	42 (20.69)	
** II**	81 (31.52)	50 (22.42)	71 (34.97)	
** III**	51 (19.84)	78 (34.98)	55 (27.09)	
** IV**	65 (25.29)	49 (21.97)	35 (17.25)	

### Analysis of lipogenic genes expression in CRC samples

To identify the genes with significant dysregulation ratio in CRC tissues, the expression pattern of 61 genes involved in lipid-metabolic assessed using the Realtime-PCR between 257 CRC samples and their matched normal samples in the training set. Considering fold-change higher than 2 as criteria, five upregulated genes including ACSL5 (2.54-fold, *P* < 0.01), ACOT8 (3.27-fold, *P* < 0.01), FASN (3.19-fold, *P* < 0.01), SCD1 (2.36-fold, *P* < 0.01) and HMGCS2 (3.38-fold, *P* < 0.05) were achieved. These lipogenic genes are involved in the transportation of lipids and activation of FAs as well as mediating the cellular signaling. Additional details are provided in S1 Table.

### Establishment of the lipogenic gene panel

Table 2 demonstrates the diagnostic performance of ACSL5, ACOT8, FASN, HMGCS2, and SCD1 as individual biomarkers for discriminating CRC tumors from the normal group. According to the data, all five genes were good predictors (AUC > 0.7), and ACSL5 achieved an AUC of 0.8131 (0.7506–0.8755).

**Table 2:** Diagnostic performance of lipogenic genes in CRC.

Gene	Sensitivity	Specificity	Youden’s index J	AUC	*p* value	95% CI
**ACOT8**	58.41	89.22	0.4763	0.7666	< 0.0001	0.6997–0.8334
**ACSL5**	68.08	87.29	0.5537	0.8131	< 0.0001	0.7506–0.8755
**FASN**	57.34	91.45	0.4863	0.7225	< 0.0001	0.6578–0.8188
**HMGCS2**	61.12	82.33	0.4345	0.7315	< 0.0001	0.6588–0.8042
**SCD1**	59.02	93.17	0.5203	0.7559	< 0.0001	0.6868–0.8249

To develop a single risk score using all five genes (ACOT8, ACSL5, FASN, HMGCS2 and SCD1), we used a previously developed strategy with regression analysis for multiple biomarkers [21]. In summary, the expression level of the genes was log_2_ transformed to reduce the variations between the value of each gene and used for generating of the logistic regression coefficients. The risk score for each sample was calculated as the sum of the risk score for each gene, which was yielded by multiplying the expression level of a gene by its corresponding coefficient (Risk score  =  ∑ logistic regression coefficient of gene *Mi* × expression level of gene *Mi*). Subjects were subsequently divided into two groups using the median cutoff risk score as a threshold.

**Fig 1:**
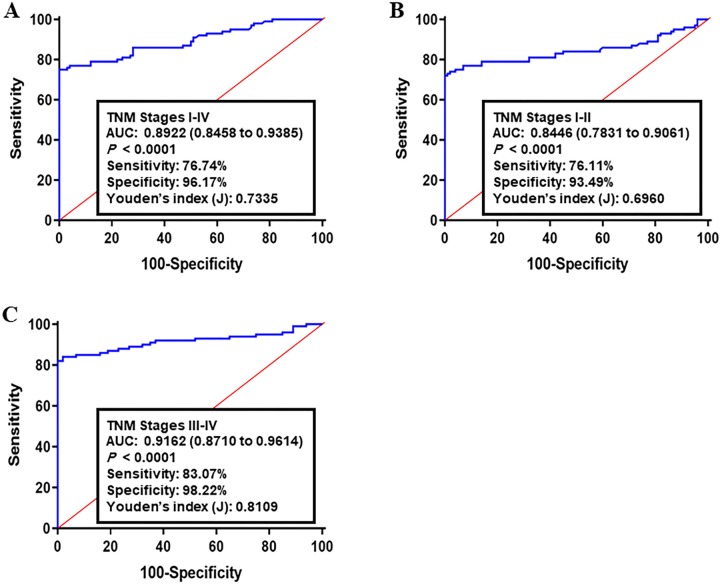
Receiver operating characteristics (ROC) curve analysis of the log *it* model with the ACOT8/ACSL5/FASN/HMGBCS2/SCD1 panel in the training set. The study group consisted of 257 CRC tumor specimens and matched adjacent normal tissues. Using the optimal cutoff value as 0.493, the diagnostic performance of gene panel for discriminating (A) All TNM stages, (B) early CRC (I-II TNM stages), and **(C)** advanced CRC (III-IV TNM stages) from healthy samples was examined. Log *it* (p) of model was 0.429 + 0.659 x ACOT8 + 0.084 x ACSL5 + 0.029 x FASN + 0.201 x HMGCS2 + 0.119 x SCD1.

To calculate the predicted point of detecting CRC by the five lipogenic genes panel, a stepwise logistic regression coefficients model was established between 257 CRC tumor specimens and matched adjacent normal tissues in the training set. The predicted estimation of being diagnosed with cancer from the log *it* model based on the five selected lipogenic genes panel (Table 3), and Log *it* (P) = 0.429 + 0.659 x ACOT8 + 0.084 x ACSL5 + 0.029 x FASN + 0.201 x HMGCS2 + 0.119 x SCD1 was used to create the ROC curve. Using the optimal cutoff value as 0.493, the training set samples were divided into two groups with high-risk and low-risk scores of colon cancer. The combination of the measurements of these genes into a single risk score based on logistic regression coefficients enhanced the accuracy of diagnosis and was higher than their values as a single biomarker, and the goodness of fit test indicated a good adjustment (Hosmer–Lemeshow test, *P* = 0.729). For all CRC stages (I-IV TNM stages), the AUC of lipogenic genes panel was 0.8922 (95% CI: 0.8458–0.9385, sensitivity: 76.74% and specificity: 96.17%, Fig 1A). For early CRC (I-II TNM stages), the AUC was 0.8446 (95% CI: 0.7831–0.9061, sensitivity: 76.11% and specificity: 93.49%, Fig 1B). For advanced CRC (III-IV TNM stages), the AUC was 0.9162 (95% CI: 0.8710–0.9614, sensitivity: 83.07% and specificity: 98.22%, Fig 1C).

**Table 3:** Logistic regression of selected lipogenic genes and lipogenic genes panel in training set.

Variable	Coefficient	Std. Error	Odd ratio	95% CI	*p* value
**ACOT8**	0.659	0.124	1.932	1.2551–2.0384	< 0.0001
**ACSL5**	0.084	0.019	1.087	0.6282–1.4491	< 0.0001
**FASN**	0.029	0.007	1.029	0.7119–1.3167	< 0.0001
**HMGCS2**	0.201	0.041	1.222	0.8375–1.8387	< 0.0001
**SCD1**	0.119	0.033	1.126	0.7298–1.5021	< 0.0001
**Constant**	0.429				

### Validation of the lipogenic gene panel

**Fig 2:**
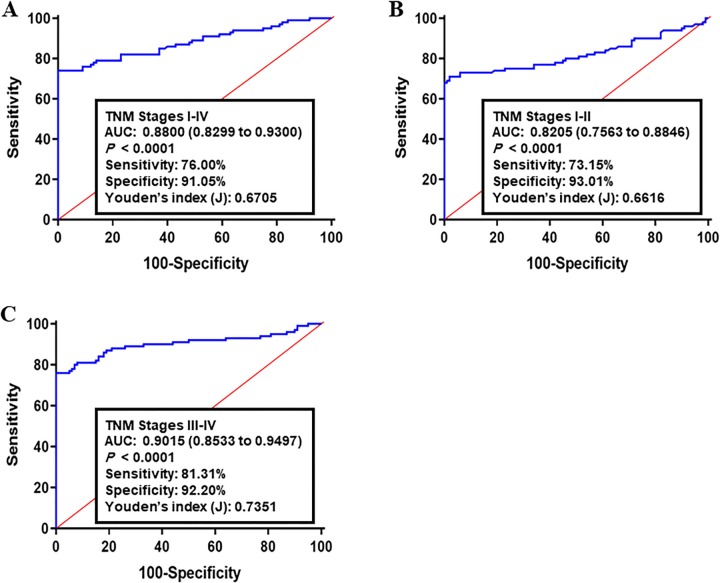
Receiver operating characteristics (ROC) curve analysis of the log *it* model with the ACOT8/ACSL5/FASN/HMGBCS2/SCD1 panel in the validation set I. The study group consisted of 223 CRC tumor specimens and matched adjacent normal tissues. Using the optimal cutoff value as 0.493, the diagnostic performance of gene panel for discriminating (A) All TNM stages, (B) early CRC (I-II TNM stages), and (C) advanced CRC (III-IV TNM stages) from healthy samples was examined. Log *it* (p) of model was 0.429 + 0.659 x ACOT8 + 0.084 x ACSL5 + 0.029 x FASN + 0.201 x HMGCS2 + 0.119 x SCD1.

The diagnostic performance of the panel was subsequently estimated in the validation set I consisted of 223 CRC tumor specimens and their matched adjacent normal tissues. The corresponding AUC for all CRCs (I-IV TNM stages) compared to healthy group was 0.8800 (95% CI: 0.8299–0.9300; sensitivity: 76.00% and specificity: 91.05%, Fig 2A). Analyzing of early CRCs resulted in an AUC of 0.8205 (95% CI: 0.7563–0.8846; sensitivity: 73.15% and specificity: 93.01%, Fig 2B). The AUC of advanced CRCs versus normal group was 0.7351 (95% CI: 0.8533–0.9497; sensitivity: 81.31% and specificity: 92.20%, Fig 2C).

**Fig 3:**
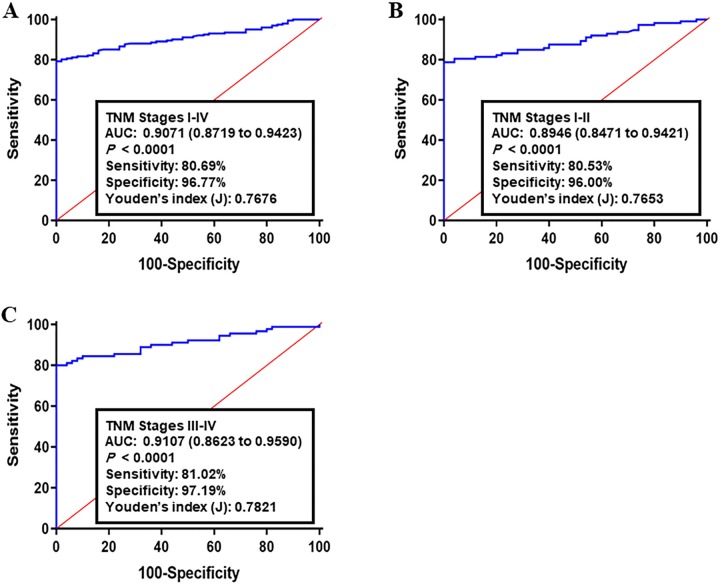
Receiver operating characteristics (ROC) curve analysis of the log *it* model with the ACOT8/ACSL5/FASN/HMGBCS2/SCD1 panel in validation set II. The study group consisted of 203 CRC tumor specimens and 50 normal tissues. Using the optimal cutoff value as 0.493, the diagnostic performance of gene panel for discriminating (A) All TNM stages, (B) early CRC (I-II TNM stages, n = 113), and **(C)** advanced CRC (III-IV TNM stages, n = 90) from healthy samples was examined. Log *it* (p) of model was 0.429 + 0.659 x ACOT8 + 0.084 x ACSL5 + 0.029 x FASN + 0.201 x HMGCS2 + 0.119 x SCD1.

To further examine the diagnostic performance of the target panel, an independent sample group consisted of 253 TCGA-COAD profiles (203 CRCs and 50 normal controls) was considered as the validation set II. The corresponding AUC for all CRCs (I-IV TNM stages) compared to healthy group was 0.9071 (95% CI: 0.8719–0.9423; sensitivity: 80.69% and specificity: 96.77%, Fig 3A). Analyzing of early CRCs resulted in an AUC of 0.8946 (95% CI: 0.8471–0.9421; sensitivity: 80.53% and specificity: 96.00%, Fig 3B). The AUC of advanced CRCs versus normal group was 0.9107 (95% CI: 0.8623–0.9590; sensitivity: 81.02% and specificity: 97.19%, Fig 3C).

### Prognostic performance of lipogenic genes in CRC

Cox’s proportional hazards model was applied to calculate the prognostic value of lipogenic genes in CRC tumors, and for a better conclusion, the clinical features and histopathological data were also considered in either univariate and multivariate analyses. According to the data obtained from the univariate analysis, TNM staging and lymph node metastasis had the best prognostic values between the three independent target groups (Table 4). Besides these clinical variables, Age higher than 70 was also considered as a non-modifiable risk factor in multivariate analysis.

**Table 4:** Univariate Cox regression analysis of overall survival of the clinical variables in CRC patients.

		Training set (n = 257)		Validation set I (n = 223)		Validation set II (n = 203)	
		HR (95% CI)	*P* value	HR (95% CI)	*P* value	HR (95% CI)	*P* value
**Gender**	**Female**						
	**Male**	1.429 (1.215–2.033)	0.73	1.927 (1.433–3.558)	0.338	1.672 (1.111–3.201)	0.532
**Age**	**≤** **55**						
	**55 < x < 70**	3.793 (3.519–4.208)	0.031	2.209 (1.997–2.747)	0.291	1.873 (1.249–3.337)	0.483
	**≥ 70**						
**Location of tumor**	**Left Colon**						
	**Right Colon**	1.693 (1.386–2.771)	0.506	2.029 (1.466–3.181)	0.199	1.755 (1.313–2.545)	0.511
	**Rectosigmoeid**						
**Differentiation**	**Well**						
	**Moderately**	2.057 (1.723–3.321)	0.216	2.376 (1.118–3.403)	0.283	2.206 (1.636–2.817)	0.249
	**Poorly**						
**TNM Stage**	**I**						
	**II**						
	**III**	3.719 (3.202–5.525)	<0.01	3.873 (3.113–5.649)	<0.01	3.679 (3.205–4.949)	<0.01
	**IV**						
**Lymph node Metastasis**	**Yes**						
	**No**	3.406 (2.999–4.116)	0.023	3.099 (2.711–3.828)	0.038	3.023 (2.888–4.049)	0.045

The prognostic value of lipogenic genes panel was examined unadjusted and together with the clinical variables (Age, TNM staging, and lymph node metastasis) by the multivariate analysis. As already shown in Table 5, there was a significant correlation between abnormal level of the ACOT8, ACSL5, FASN, HMGCS2, and SCD1 genes in the form of a panel and worse OS in training set (HR (95% CI): 4.869 (4.523–6.302), *P* <0.001), validation set I (HR (95% CI): 4.803 (4.530–6.221), *P* <0.001), and Validation set II (HR (95% CI): 4.855 (4.339–6.029), *P* <0.001). The multivariate analysis also introduced the lipogenic genes signature as an independent prognostic indicator for 5-years OS with about 5-fold higher risk of tumor recurrence for CRC patients in training set (HR (95% CI): 4.869 (4.523–6.302), *P* <0.001), validation set I (HR (95% CI): 4.803 (4.530–6.221), *P* <0.001), and in the validation set II (HR (95% CI): 5.117(4.814–5.772), *P* <0.001, Table 5).

**Table 5:** Univariate and multivariate cox regression analysis of overall survival of lipogenic genes signature as individual biomarkers and in the form of a panel in CRC patients.

	Training set (n = 257)				Validation set I (n = 223)				Validation set II (n = 203)			
	Unadjusted		Adjusted		Unadjusted		Adjusted		Unadjusted		Adjusted	
	HR (95% CI)	*P* value	HR (95% CI)	*P* value	HR (95% CI)	*P* value	HR (95% CI)	*P* value	HR (95% CI)	*P* value	HR (95% CI)	*P* value
**ACOT8**	4.393		3.624		4.819		3.725		4.921		3.737	
**High vs. Low**	(4.007–5.228)	<0.001	(3.016–4.099)	<0.01	(4.402–6.193)	<0.001	(3.293–4.183)	<0.01	(4.255–6.708)	<0.001	(3.333–3.455)	<0.01
**ACSL5**	4.246		3.033		4.028		2.888		3.492		3.039	
**High vs. Low**	(4.067–5.009)	<0.001	(2.828–3.663)	<0.01	(3.903–4.878)	<0.001	(2.577–3.467)	0.069	(2.733–3.671)	0.02	(2.603–3.552)	<0.01
**FASN**	3.509		3.337		3.434		3.122		3.367		3.059	
**High vs. Low**	(2.777–4.483)	<0.01	(2.709–4.283)	0.028	(2.837–4.558)	0.019	(2.679–4.371)	0.038	(2.729–4.228)	0.026	(2.893–4.071)	<0.01
**HMGCS2**	4.122		3.846		3.957		3.772		4.008		3.843	
**High vs. Low**	(3.439–4.492)	<0.001	(3.368–4.132)	<0.01	(3.652–4.448)	<0.01	(3.442–4.335)	<0.01	(3.594–4.543)	<0.001	(3.777–4.483)	<0.01
**SCD1**	4.082		3.869		4.003		3.803		4.117		3.855	
**High vs. Low**	(3.528–4.411)	<0.001	(3.523–4.302)	<0.01	(3.587–4.808)	<0.001	(3.530–4.221)	<0.01	(3.814–4.772)	<0.001	(3.339–4.029)	<0.01
AJCC Risk*	2.936				3.601				3.462			
**High vs. Low**	(2.293–6.993)	0.335			(2.483–5.002)	<0.01			(2.338–8.983)	0.021		
**Gene Panel**	5.082		4.869		5.003		4.803		5.117		4.855	
**High vs. Low**	(4.528–6.411)	<0.001	(4.523–6.302)	<0.001	(4.587–6.808)	<0.001	(4.530–6.221)	<0.001	(4.814–5.772)	<0.001	(4.339–6.029)	<0.001

**Fig 4:**
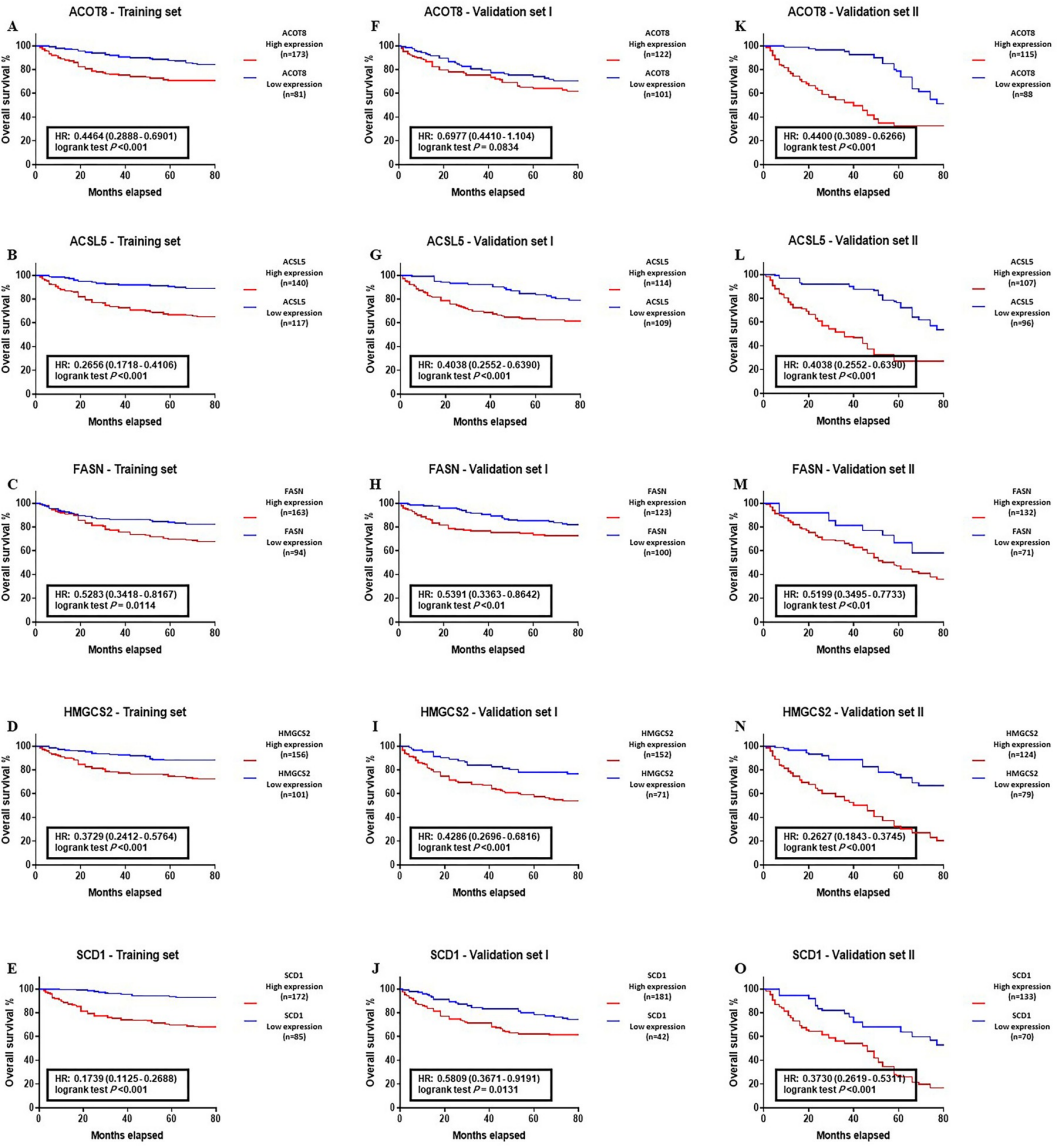
Correlation between the abnormal expression of ACOT8, ACSL5, FASN, HMGCS2, and SCD1 genes and overall survival (OS) of colon cancer patients. (A-E) Training group, (F-J) Validation I group, and (L-O) Validation II group. Kaplan-Meier analysis indicated a reverse correlation between individual genes upregulation and poor survival of CRC patients. (Training group n = 257, Validation group I n = 223, Validation group II n = 203.

**Fig 5:**
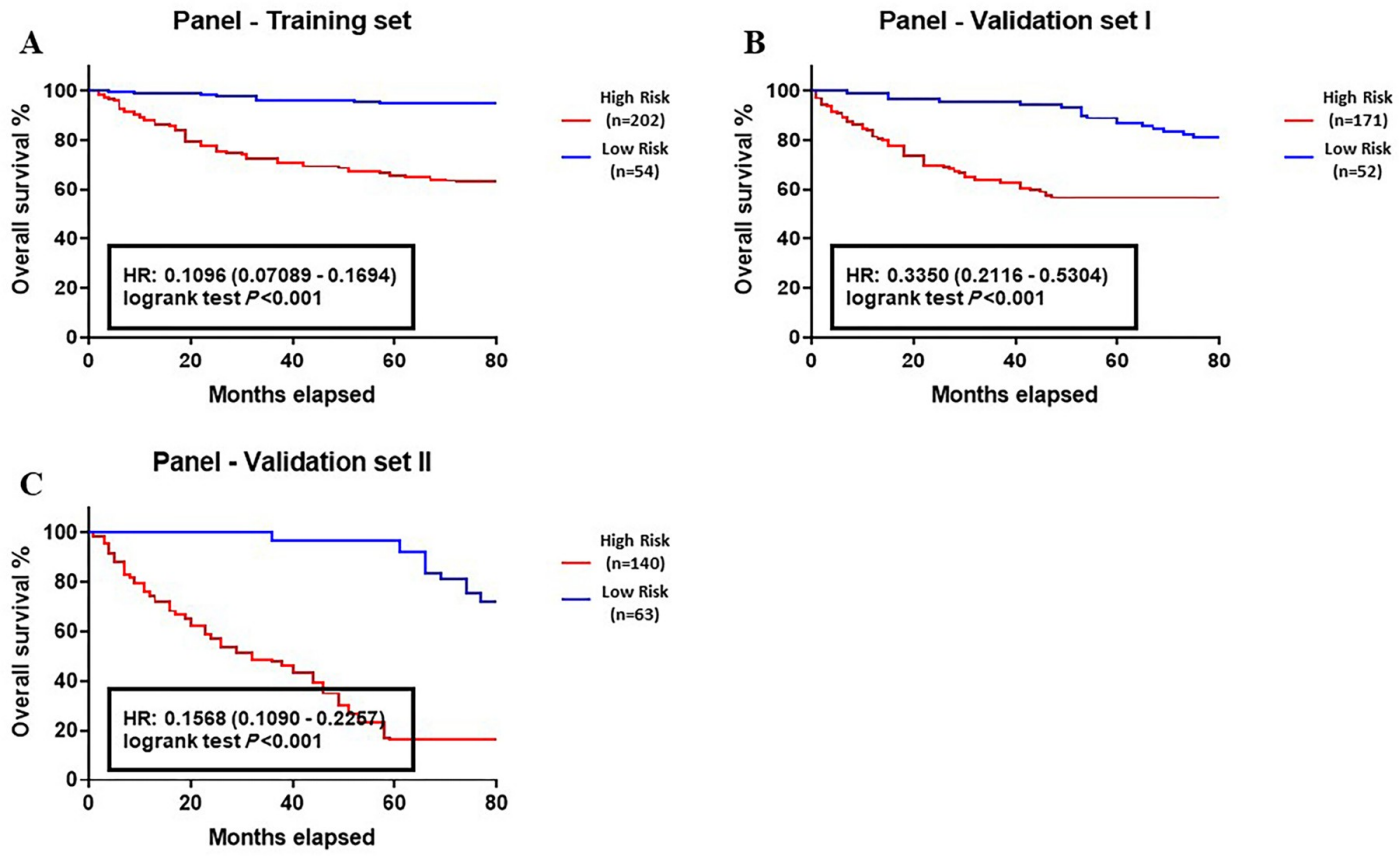
Correlation between the expression of lipogenic genes panel and overall survival (OS) of colon cancer patients. (A) Training group, (B) Validation group I, and (C) validation group II. OS analysis of lipogenic genes panel showed a statistical correlation between the high predicted point of genes panel and worse OS of CRC patients. (Training group (n = 257): Low risk n = 54, High risk n = 202; Validation group I (n = 223): Low risk n = 52, High risk n = 171; Validation group II (n = 203): Low risk n = 63, High risk n = 140).

Kaplan–Meier survival analysis was carried out to estimate the 5-year OS of CRC population with the abnormal expression of individual genes (Fig 4) and lipogenic genes panel (Fig 5). The median follow-up and 5-year OS of patients in target groups were as follows: Training set = 72 months and 87.4%, respectively; Validation set I = 75 months and 89.1%, respectively, and Validation set II = 77 months and 85.5%, respectively. The results obtained from the analysis of individual genes in training set yielded a HR (95% CI): 0.4464 (0.2888–0.6901), *P* < 0.001 for ACOT8, HR (95% CI): 0.2656 (0.1718–0.4106), *P* < 0.001 for ACSL5, HR (95% CI): 0.5283 (0.3418–0.8167), *P* = 0.0114 for FASN, HR (95% CI): 0.3729 (0.2412–0.5764), *P* < 0.001 for HMGCS2, and HR (95% CI): 0.1739 (0.1125–0.2688), *P* < 0.001 for SCD1 (Fig 4A–4E). In the validation set I, the results were HR (95% CI): 0.6977 (0.4410–1.104), *P* = 0.0834 for ACOT8, HR (95% CI): 0.4038 (0.2552–0.6390), *P* < 0.001 for ACSL5, HR (95% CI): 0.5391 (0.3363–0.8642), *P* < 0.01 for FASN, HR (95% CI): 0.4286 (0.2696–0.6816), *P* < 0.001 for HMGCS2, and HR (95% CI): 0.5809 (0.3671–0.9191), *P* = 0.0131 for SCD1 (Fig 4F–4J). The analysis of validation set II showed a HR (95% CI): 0.4400 (0.3089–0.6266), *P* < 0.001 for ACOT8, HR (95% CI): 0.3764 (0.2622–0.5403), *P* < 0.001 for ACSL5, HR (95% CI): 0.5199 (0.3495–0.7733), *P* < 0.01 for FASN, HR (95% CI): 0.2627 (0.1843–0.3745), *P* < 0.001 for HMGCS2, and HR (95% CI): 0.3730 (0.2619–0.5311), *P* < 0.001 for SCD1 (Fig 4L–4O). These data indicated that as an independent prognostic factors, the abnormal level of ACOT8, ACSL5, FASN, HMGCS2, and SCD1 is correlated with the worse clinical outcome of the CRC patients.

Kaplan-Meier analysis of lipogenic genes panel also showed a statistically correlation between high predicted point of genes panel and worse OS of CRC patients is training set (HR (95% CI): 0.1096 (0.07089–0.1694), *P* < 0.001, Fig 5A), validation set I (HR (95% CI): 0.3350 (0.2116–0.5304), *P* < 0.001, Fig 5B), and validation set II (HR (95% CI): 0.1568 (0.1090–0.2257), *P* < 0.001, Fig 5C). These data demonstrated that the combination of lipogenic genes yielded a higher prognostic value and accuracy than the other clinical features used in this study.

## Discussion

Nowadays, the histopathological diagnosis of CRC is carried out with the TNM classification system. However, due to the lack of prediction accuracy via TNM staging, especially in early CRC cases, the time needed to use a proper therapeutic strategy may be lost. Thus, besides improving the outcome prediction for early CRC, the identification of new biomarkers with higher sensitivity and specificity will pave the road for choosing the best treatment with less cost and risks for CRC patients.

The prognostic value of various gene expression signatures has been investigated in CRC during the last decade. For example, an Oncotype DX assay of 12 genes involved in cell cycle control, stromal response and genotoxic stress, indicated a significant association between recurrence score (RS) and risk of deficiency in mismatch repair (MMR) along with tumor recurrence in over 1700 stage II CRC patients [22]. Accordingly, RS was reported as an independent predictor of CRC recurrence, particularly for cases with 3 MMR-I tumors [22]. The data was in line with an earlier report of 1,436 stage II CRC patients in which RS was significantly correlated with the risk of recurrence beyond the traditional clinical features [23]. An optimal set of 18-gene coloprint assay divided the I-III stages CRC tumors (n = 188) into low and high risks disease groups [24]. According to the data, the panel was succeeded in identifying low-risk cancer cases with a significantly higher 5-year relapse-free survival rate compared to the rest of patients (87.6% vs. 67.2%, *P* < 0.05). Profiling of a 23-gene ColoGuideEx panel between Dukes'B CRC patients (n = 72) indicated an OS accuracy of 78% (sensitivity: 72% and specificity: 83%), and statistically disease-free time difference (*P* < 0.0001) between the predicted relapse and disease-free patients [25]. On the other hand, Agesen et al. validated a ColoGuideEx panel consisted of 13 genes for tumor relapse prediction in patients with stage II CRC [26]. In another study, a 32,000 cDNA microarray analysis was performed to identify molecular markers for accurate CRC staging [27]. The authors optimized a 43-gene set with an improved prediction capability of 3-year OS than Dukes' staging (*P* < 0.05) and announced their molecular staging classifier more accurate than the traditional clinical staging [27]. Although these reports are promising, however, suggested gene panels consisted of intracellular signaling mediators with a minor biological significance, and through that might affect the interpretation of the results. Therefore, focusing on the main biological processes such as cellular metabolism with a high impact on cancer initiation and progression may introduce potential biomarkers for CRC screening along with new therapeutic strategies and targets.

**Fig 6:**
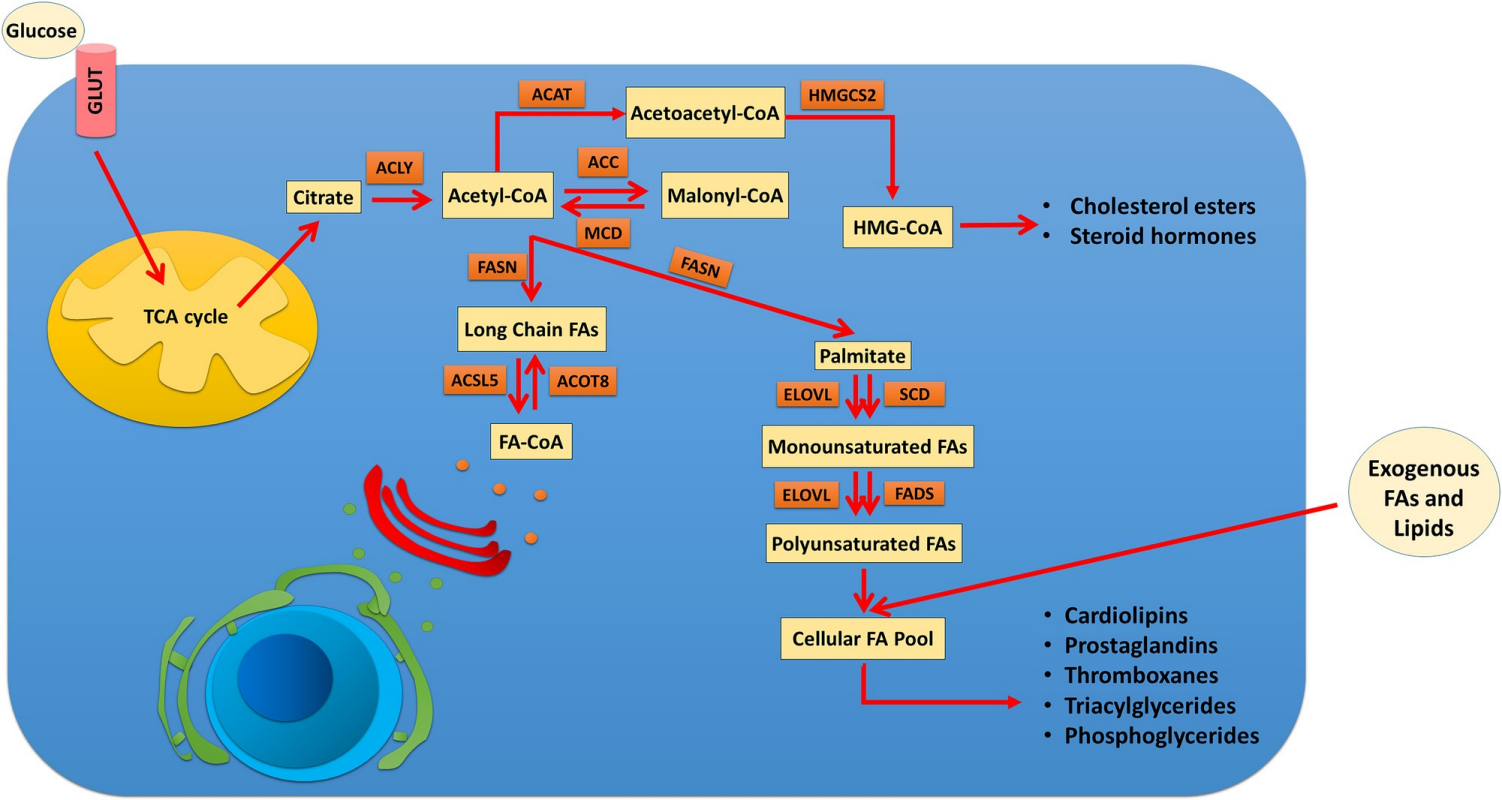
Schematic view of the roles of ACOT8, ACSL5, FASN, HMGBCS2, and SCD1 genes in lipid metabolism.

Dysregulation of cellular signaling pathways is one of the important hallmarks of cancers. Considering this point that abnormal activity of energy metabolism cascades such as lipid metabolism has a distinctive role in cancer development, their expression and activity status has been subjecting of interest of researchers for screening and therapeutic inventions. In line with previous studies, we examined the putative correlation between the lipogenic genes signatures and the prediction of the outcome of early CRC patients. Analysis of three independent cohorts indicated significant upregulation of ACOT8, ACSL5, FASN, HMGCS2, and SCD1 as the key dysregulated metabolic factors within the study population (Fig 6). Acyl-CoA Thioesterase 8 (ACOT8) is a peroxisomal lipolysis‑related enzyme catalyzing fatty acyl‑CoA breakdown into FFA and COA molecules for β-oxidation. The potential role of ACOT8 in cancer development has been raised regarding the reports of its overexpression in hepatocellular carcinoma and ovarian cancer cells[28, 29]. Meanwhile, the possible prognostic role of ACOT8 has only investigated during lymph node metastasis of lung adenocarcinoma in which the authors reported that ACOT8 upregulation was associated with poorer prognosis of lung cancer patients [30]. ACSL5 is a member of Acyl-CoA synthetase long-chain family that unlike the other members of ACSL family, provokes β-oxidation [31] or triacylglycerols storage [32] due to its cellular location. ACSL5 dysregulation was previously reported in bladder cancer [33], breast cancer [33, 34], glioma [35], glioblastomas [36], and pancreatic ductal adenocarcinoma [37]. Meanwhile, the expression status of ACSL5 in CRC tumors is vague. While some studies reported that ACSL5 downregulation is associated with tumor development [33, 38] or early tumor recurrence [39], the other investigations indicated that ACSL5 overexpression plays a key role in colon cancer cells aggressiveness [40, 41]. FASN was perhaps the most studied member of our panel in oncology, which catalyzes palmitate synthesis by the condensation of malonyl-CoA and Acetyl-CoA. Downregulation of FASN with the RNAi technology has a significant impact on lipid metabolism depression and TG storage of human lymph node metastatic lesion of prostatic adenocarcinoma (LNCaP) cells [42]. Considering this point that tumor cells survival is mostly depended on FASN-mediated *de novo* synthesis of FAs, targeting the FASN enzyme is suggested as a suitable therapeutic strategy for human cancers [43]. The other target gene with a rate-limiting role in lipid metabolism was the mitochondrial 3-hydroxy-3-methylglutaryl-CoA synthase (HMGCS2). Since cancer cells use the ketogenesis as an alternative energy source, constitutive expression of HMGCS2 as the first step of this chain is essential for tumor development [11, 44]. HMGCS2 may promote the metastasis of CRC and oral cancer cells in a ketogenesis enzymatic-independent manner via HMGCS2/PPARα/Src axis activation [45]. The last member of our panel was Stearoyl-CoA-desaturase 1 (SCD1), a key enzyme downstream of FASN, which is highly activated in palmitate—monounsaturated FAs transformation by catalyzing Δ9 position desaturation [46]. SCD1 expression is reported to stimulate following activation of PI3K-Akt-mTOR pathway in cancer cells [47] and therefore has been investigated as a therapeutic target in a variety of human cancers including colon [48, 49], endometrial [50], glioblastoma [51], lung [52], and renal cell carcinoma [53].

Our study highlights the impact of ACOT8, ACSL5, FASN, HMGBCS2, and SCD1 genes in lipid metabolism along with their distinctive role in cancer initiation and progression. To our knowledge, the combined expression signature of these genes has not been investigated in human cancers until recently, and our study is the first to report this expression pattern profile in cancer tumors. Besides the announced diagnostic and prognostic values of the target panel for early CRC, our investigation provided evidence indicating CRC tumors may benefit of ACOT8/ACSL5/FASN/HMGBCS2/SCD1 axis activation for their structural and energetic demands without common lipidic toxicity such as overproducing endogenous ceramide by inhibition of SCD1 enzyme which previously reported in CRC cells [48].

To have a complete insight, this investigation analyzed the possible dysregulation in the wide range of lipogenic genes in CRC tumors. This large sample size (n = 683) allowed us to examine early and advanced CRC samples together for better visualization and to determine the sensitivity and specificity of selected biomarkers. However, there were some limitations to this study. First, including all four tumor stages made the samples collection process non-randomized and non-blinded. Also, since samples were gathered in 6 years, not all of the patients had been tested for their cholesterol level and Body Mass Index (BMI), which could help us for a better conclusion.

## Conclusion

Taking together, our panel demonstrates a better prognostic performance for the screening of the early CRC and tumor recurrence compared to the validated clinical risk scales by the American Society of Clinical Oncology (ASCO). However, further investigations are needed to elucidate the mechanisms involved in ACOT8/ACSL5/FASN/HMGBCS2/SCD1 axis activation.

## Supporting information

S1 Table The expression level analysis of 61 lipogenic genes between CRC tumors and matched adjacent normal tissues in the training set (n = 257). (XLSX)
